# Surface Modification of Bacterial Cellulose for Biomedical Applications

**DOI:** 10.3390/ijms23020610

**Published:** 2022-01-06

**Authors:** Teresa Aditya, Jean Paul Allain, Camilo Jaramillo, Andrea Mesa Restrepo

**Affiliations:** 1 Ken and Mary Alice Lindquist Department of Nuclear Engineering, Pennsylvania State University, University Park, PA 16802, USA; allain@psu.edu (J.P.A.); cxj5289@psu.edu (C.J.); 2 Department of Biomedical Engineering, Pennsylvania State University, University Park, PA 16802, USA; aqm6463@psu.edu; 3 Materials Research Institute, Pennsylvania State University, University Park, PA 16802, USA; 4 Institute for Computational and Data Sciences, Pennsylvania State University, University Park, PA 16802, USA; 5 Huck Institutes of the Life Sciences, Pennsylvania State University, University Park, PA 16802, USA

**Keywords:** bacterial cellulose, surface chemistry, surface analysis, surface functionalization, interface, tissue engineering, bactericidal

## Abstract

Bacterial cellulose is a naturally occurring polysaccharide with numerous biomedical applications that range from drug delivery platforms to tissue engineering strategies. BC possesses remarkable biocompatibility, microstructure, and mechanical properties that resemble native human tissues, making it suitable for the replacement of damaged or injured tissues. In this review, we will discuss the structure and mechanical properties of the BC and summarize the techniques used to characterize these properties. We will also discuss the functionalization of BC to yield nanocomposites and the surface modification of BC by plasma and irradiation-based methods to fabricate materials with improved functionalities such as bactericidal capabilities.

## 1. Introduction

Cellulose is one of the most abundant materials on earth and have recently gained widespread interest in several technological applications because it is a renewable, sustainable, eco-friendly, and biocompatible material [1,2,3,4]. Cellulose has become a popular choice for intensive ongoing research and a keen interest has developed on the emerging applications of robust and translucent cellulose and their advanced functionalities in electronics, photonics, energy storage, wearable or injectable device [2,5,6]. Bacterial cellulose (BC) is a polysaccharide (C_6_H_10_O_5_)_n_, with characteristic microstructures and is derived from microorganisms such as, Gram-negative bacterial species of the genera *Gluconacetobacter*, *Sarcina*, *Azobacter Achromobacter*, *Aerobacter*, *Salmonella*, *Rhizobium*, *Pseudomonas* and *Alcaligenes*, as well as oomycetes and green algae [7,8,9]. The serendipitous discovery of BC during vinegar fermentation by A. J. Brown in 1886, marked the beginning of study in this field and it was not until later, being a natural nanomaterial, its application in the biomedical field was explored [10]. Cellulose can also be synthesized from cell-free systems from a single cell line using a cell-lysing technique and employing cellulose synthesizing enzymes [11,12,13,14].

Despite its numerous possibilities of application, the key challenge of BC remains in the frequent occurrence of bacterial adhesion, growth and eventually infection in these artificial membranes. Integration of functionalized biomaterial is challenging specially to assimilate properties of the material for advanced biomedical applications. While a popular line of research continues around combining metal/metal chalcogenide nanoparticle and polymer materials, cellulose with appropriate functionalization remains a popular choice for such biocompatible polymer composites. Compared to other types of cellulose, BC possesses biocompatibility and mechanical properties that make it an ideal material for biomedical applications [15,16]. Moreover, BC possesses high purity with high water retaining capacity, easily modifiable biodegradability, biocompatibility with facile production, purification and mouldability. The micromorphology of BC has a distinctive network of cellulose nanofiber consisting of unique feature rendering them as oil absorbents, fuel cell, catalyst and biomedical applications such as drug delivery, tissue engineering, skin repair and wound dressing [17,18,19,20,21,22,23,24].

BC has become a popular choice with research in emerging applications in both tissue engineering and regenerative medicine. Tissue engineering and regenerative medicine, attempt to create functional human tissues from cells by repairing or replacing tissue in organs which may fail due to disease, genetic errors, congenital abnormalities, or traumatic injury [24,25]. While tissue engineering encompasses the scaffolds and growth factors which affects the regeneration or replacement of damaged tissues, regenerative medicine circumscribes cell therapy, gene therapy and immunomodulation to encourage tissue or organ repair or reconstruction inside the body. The important factors for successful tissue engineering are right cells for the tissue, the right environment such as the scaffold to support the cells, the right biomolecules such as growth factor to make cells healthy and productive, along with physical and mechanical forces to influence the development of the cells. In some cases, the designed scaffold dissolve overtime while in others they remain to provide lifelong strength, endurance and support to the system which mimic the organs. Damage to blood vessel walls also known as aneurysms can be a critical trauma leading to high fatality rates [24]. Clipping and endovascular coiling by surgery are the conventional treatment for such conditions with the serious risk of thrombus formation as post-surgical complication. Apart from longevity of the mechanical characteristics, blood vessel grafts or implantation using natural tissue source from animals or polymers has been employed in many cases with the risk of immunological rejection, or activation of contact coagulation system. Blood vessel tissue regenerative reconstruction of functional human tissue is the state-of-the-art cure, and an avenue which has gained tremendous momentum over time with the help of nanotechnology. The aim of tissue engineering is to create a three-dimensional (3D) cell-biomaterial environment which will mimic tissue/organ.

Surface functionalization of BC is a crucial step rendering the material more active and efficient. The goal of this review is to discuss the structure and properties of BC, functionalization of BC via chemical and physical means and describe the biomedical application of functionalized BC hydrogels, specifically for vascular and neural applications, wound healing, and bactericidal interfaces.

## 2. Structure and Properties of BC

**Figure 1:**
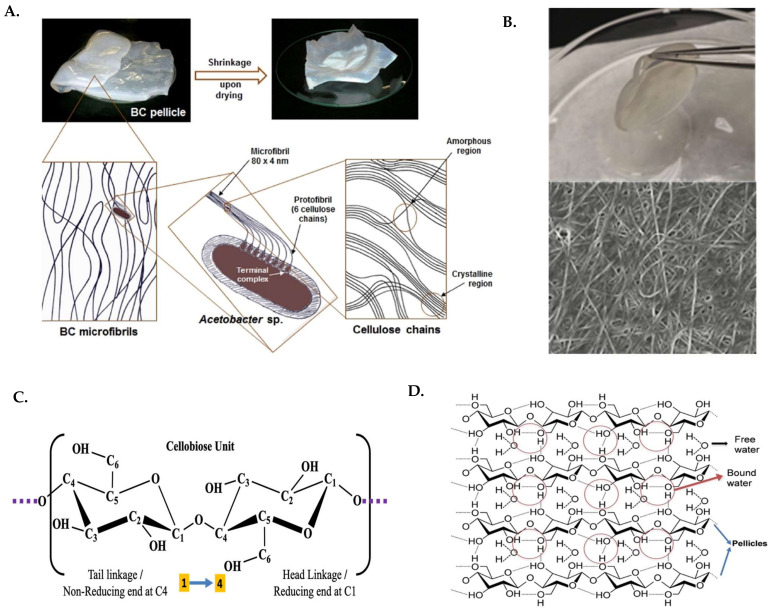
(**A**) Arrangement of microfibril in amorphous and crystalline region and its macroscopic appearance in wet conditions. (**B**) BC loaded with water and its SEM image. (**C**) Molecular structure of “cellobiose unit.” (**D**) H-bonding in the matrix of the BC. Reproduced with permission from [27] (Copyright © 2022, Elsevier), [32] (Open Access).

Among the different microbial origins of BC, those produced by the stationary culture of *Gluconacetobacter xylinus* (*G. xylinus*), formerly known as *Acetobacter xylinum* (*A. xylinum*), in laboratory settings of air-liquid interphases, results in tunable films or pellicles with thickness controlled by the days they are allowed to stand [9,21,26]. BC possesses a gelatinous consistency made of an indefinite length of randomly interwoven microfibrils with ribbon-like appearance and consisting of polymerization of the cellobiose dimer (Figure 1A,B). *G. xylinus*, a Gram-negative bacterium, forms cellulose microfibrils by polymerization of the available glucose in the culture medium and their subsequent crystallization [27]. It has been discovered that the nematic ordered cellulose substrate, where the liquid crystals have molecules which are parallel but not in well-defined planes, are the driving factor for the controlled direction of fibers secretion, while the different kind of -OH group at C2, C3 and C6 in the cellobiose differ in their polarity and thereby in the molecular H-bonding and van der Waals interactions (Figure 1C) [28,29]. The biosynthesis of BC from bacteria involves cellulose chains which are polymerized by cellulose synthases A (CesA) from activated glucose. These single chains release through the bacterial extracellular membrane by rosette terminal complexes [30]. The cell membrane pore extrudes glucan chains and distribute them over the cell envelope. In this process, β-glycosidic linkages bind D-(1→4) anhydro glucopyranose units, giving place to polymeric chains (Figure 1C,D). The macromolecules hence organize in units producing subfibrils of 10–15 glucan chains that further assemble to form microfibrils, and finally microfibril bundles with diameter of 20–100 nm forms a gelatinous membrane. The microfiber diameter determines the properties and applications of the BC film [19]. Small diameters provide BC with high surface area for the interaction with biological molecules, and their characteristic stress strain nature closely mimic the inherent complexity and hierarchical structure of native tissues [31].

Some of the diverse parameters which affects the formation of network in BC are (a) environmental factors such as temperature, pH, dissolved oxygen, (b) speed of stirring or agitation of the growth medium (c) cultivation time and (d) conditions of the cultural media conditions, like the carbon and nitrogen sources, nutrients for the growth of microorganism and the presence of various additives. The structural singularity of the BC fibrillated 3D network produces distinct mechanical properties, with high degree of crystallinity (60–80%) and a Young’s modulus of 15–30 GPa, one of the highest of all two-dimensional (2D) organic material. The high aspect ratio of the fibers provide a high surface area and remarkable liquid loading capacity of up to 99 wt.% [30]. These loose bundles form cellulose ribbons comprised of about 1000 polyglucan chains. Continuous spinning of cellulose ribbons forms a highly pure 3D structure of nanofibers stabilized by inter- and intra-fibrillar hydrogen bonds (Figure 1D) [30]. During crystallization, β-1,4-glucan chains via intra and intermolecular hydrogen bonds, and hydrophobic and van der Waals interaction forms subsequent stacking and thickening of the parallel chain (Cellulose I) in the extracellular space [32,33]. This BC treated with around 7–16.5 wt.% NaOH during mercerization process, formed more stable anti parallel chains (Cellulose II). Their intimate H-bonds give rise to a 3D arrangement more energetically favorable [28]. There is also a Cellulose III and Cellulose IV, which can be prepared chemically from type I. It has been reported that Cellulose III can be produced from the native cellulose by treating it with ethylene diamine, while Cellulose IV can be obtained with hydrothermal treatments at high temperature above 100 °C [27,28,29,34,35].

Although the molecular formula of cellulose from plant origin is similar to BC, the latter has a crystalline nano-fibrillar structure that can provide a large surface area to retain large amount of liquids [9]. Native cellulose is composed of two distinct crystalline phases having same conformation but different crystal structure with different unit cell such as, I_α_ and I_β_, both of which have similar intra-chain hydrogen bonding but differ in their inter-chain hydrogen bonding. I_α_ consists of one chain in the triclinic unit cell of the cellulose crystal with the neighboring cellulose-sheets regularly displaced from one another in the same directions, and I_β_ contains two chains in each monoclinic unit cell of the cellulose crystal with cellulose-sheets of staggered arrangement [28,29,34,35]. Depending on the source of the cellulose the proportion of I_α_ and I_β_ differs. Generally, I_α_ is higher in bacterial and algal cell walls whereas I_β_ is more in cell wall of higher plants, hence that is an amount inherited from the type of cells produced.

BC characterization is an important part of assessment of their potential properties. The mechanical properties of cellulose and their important characteristic information has been reported after detailed investigation [22]. The strength and hardness of BC is corroborated by the hardness of the base hydrogel scaffold, the degree of orientation of the fibers, the volume fraction, size, porosity, density, and morphology. Some of the conventional techniques have been discussed.

**Figure 2:**
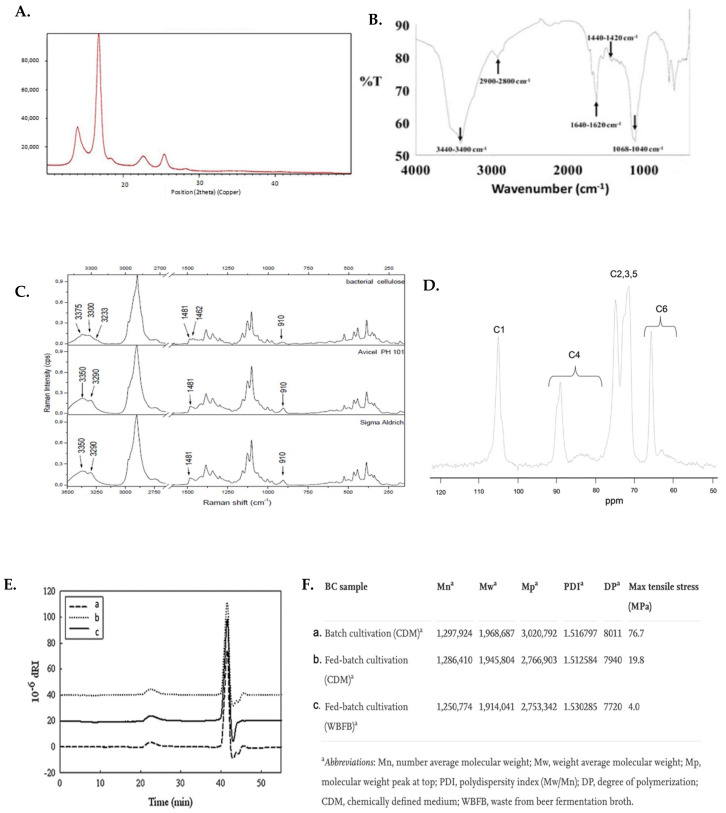
(**A**) X-ray diffraction spectrum (**B**) FTIR spectrum (**C**) Raman Spectra comparing BC, Sigma Aldrich and Avicel PH10 samples [29]. (**D**) CPMAS ^13^C-NMR spectrum of BC fully ^13^C labeled obtained without treatment and its carbon signal assignment. (**E**) GPC analysis of BC samples produced via batch cultivation in chemically defined medium (a), fed-batch cultivation of chemically defined medium (b), and fed-batch cultivation of waste from beer fermentation broth (WBFB) (c) in static conditions in a Jar fermenter, (**F**) table showing the molecular weight distribution obtained from GPS. Reproduced with permission from [38] (Copyright © 2022, Springer Science Business Media B.V., part of Springer), [39] (Copyright © 2022, Elsevier), [36] (Copyright © 2022 Elsevier).

BC has mostly ordered structure called crystalline region which shows sharp diffraction peaks, and some disordered area called the amorphous regions which forms scattered peaks (Figure 1A) which can be detected in their chemical state by X-ray diffraction spectroscopy (XRD) between 10–35° (Figure 2A). BC synthesized under different reaction conditions consists of both I_α_ and I_β_ cellulose phase in various proportions [36]. As observed, there were three distinct peaks at 2θ angles of 14.05°, 16.77° and 22.68° which is characteristic of (101), (101¯) and (002) planes of Cellulose I type. From these peaks crystallinity can be calculated, and it was observed that factors like cultivation method, carbon source, pH of the medium, stirring rate, temperature, time of fermentation and drying techniques are responsible for the percentage of crystallinity. Commercial microcrystalline cellulose is found to have low crystallinity where crystal size is measured using full width half maxima (FWHM) of the peak at 22.7° [17,18,36,37]. XRD can provide information on microstructural changes in BC which is dependent on the crystallinity, I_α_ and hydrogen bonding among the molecules. It has been reported that cellulose I_α_ and cellulose I_β_ mass fraction, crystallite size and crystallinity index is affected by cultivation mode and not by culture medium. However, crystallite size and crystallinity index is significantly affected by culture medium as well [36].

Fourier transform infra-red (FTIR) spectroscopy can identify characteristic bands of the functional groups present in the molecular structure of BC. The fingerprint region (Figure 2B) is considered between 800–1200 cm^−1^ which is specific for different polysaccharides and helps in identifying them. The characteristic O–H stretching vibration was observed around 3400–3440 cm^−1^, methylene stretching vibration of C–H at 2800–2900 cm^−1^, C–H bending vibration around 1433–1456 cm^−1^, 1045–1067 cm^−1^, C–O–C and C–O–H stretching vibration of glucose ring around 1040–1068 cm^−1^, carboxyl groups of (CO–OH, CO–NH) of the attached residual bacterial cells and protein are visible at 1600–1640 cm^−1^,and carbonyl groups (C=O) at 1420–1440 cm^−1^ can be designated. Some other peaks which may be visible are at 2489 cm^−1^ for O–D stretching and 885 cm^−1^ for N–H out of plan bending, from any carbonyl amide group that may stick as residual debris [36,38]. A plethora of information is available from the Raman spectroscopy of amorphous BC in comparison to microcrystalline cellulose obtained from higher plants, which can be divided into two regions in the range of 3500–2600 cm^−1^ and the other 1600–150 cm^−1^ (Figure 2C). The peak below 1600 cm^−1^ is indicative of the conformation of the backbone of cellulose. Below 1500 cm^−1^ the peaks belong to the internal motion of the methylene groups. The region between 150–550 cm^−1^ is assigned to the skeletal bending modes of CCC, COC. OCC, OCO bonds. This also includes methane bending of CCH, COH and the CC, CO group movement inside the units of glucopyranose ring. The peaks at 171 and 258 cm^–1^ are indicative of COH bending [29]. Between 800–1180 cm^−1^ CC and CO stretching bonds and some HCC and HCO bending can be observed. The region from 1180 and 1270 cm^−1^ belongs to the bending of HCC, HCO and COH. Around 1270–1350 cm^−1^ lies one bending of HCC and HCO, and at 1350–1430 cm^−1^ lies one bending of COH. The peaks in the range 1430 and 1500 cm^−1^ can be assigned to HCH bending. Among two peaks around 1462 and 1481 cm^−1^, higher intensity of the latter peak indicates a higher degree of crystallinity. However, the peak at 910 cm^−1^ indicates the size of the crystallites, and with increase in amorphous nature this peak shifts to lower value. The region of Raman spectra above 2700 cm^−1^ corresponds to the hydrogen bonding [29]. Two characteristic band appears, a very intensive sharper peak around 2900 cm^−1^ which refers to CH stretching vibrations and a broader one encompassing 3200–3500 cm^−1^ which indicates OH stretching vibrations. This region is very similar for both microcrystalline and amorphous cellulose and is indicative of different proportions of cellulose allomorphs I_α_ and I_β_. However, the peaks at 3350 and 3290 cm^−1^ cans be boiled down to the presence of different types of H-bonding. The lower frequency shoulder around 3200 cm^−1^ is indicative of I_α_ which is higher in case of BC while the higher frequency is indicative of I_β_ from microcrystalline cellulose. It has been studied widely that there seems to be no information in the range of 1500–2500 cm^−1^ for cellulose [36].

Cross-polarization magic-angle sample spinning ^13^C nucleic magnetic resonance (CP-MAS 13CNMR) shows the presence of six types of carbon (Figure 1B). The peaks obtained are at 105, 88, 76, 73, 71, 65 ppm (Figure 2D). The chemical environment of the BC molecules can be understood from the intensity and position of the peaks [36,39]. The polymerization of BC is a crucial chemical reaction which can be investigated using Gel permeation chromatography (GPC) using weight method with an eluent like tetrahydrofuran. While the larger molecules elute in the initial phase of the elution time, the smaller particles are removed towards the end as was for BC (Figure 2E). From a high-performance GPC system vital information can be furnished on, Weight average molecular weight (Mw), Number average molecular weight (Mn), polydispersity index (PDI = Mw/Mn) and with the knowledge of the monomer molecular weight (MW), the degree of polymerization (DP = Mn/MW) for the BC polymer can be obtained which governs the physical properties (Figure 2F) [36,40]. In each case the BC synthesized showed similar pattern but differed in the position of the peak. Analyzing the peaks around 22 ppm the DP of batch cultivation using chemically defined medium signified more aggregation of the polymers, hence a high molecular weight and thereby also a high mechanical strength. The Polydispersity index (PDI) around 1.5 signifies a uniformity in the size of the polymers formed.

**Figure 3:**
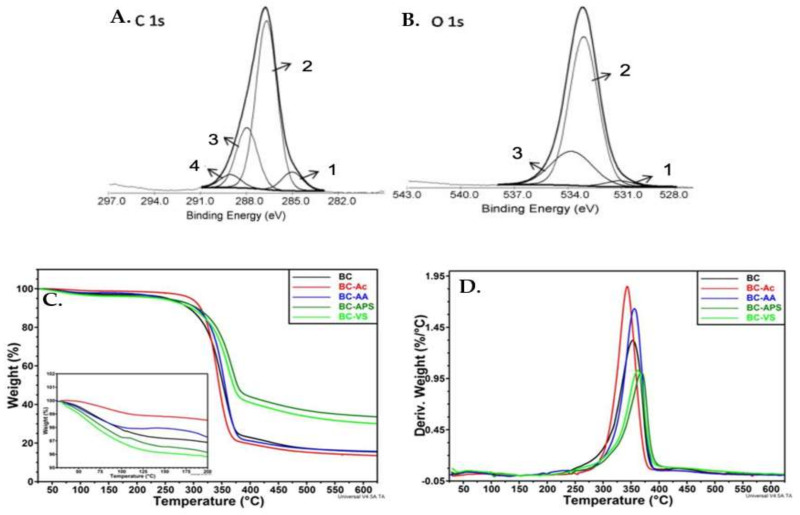
(**A**,**B**) High Resolution XPS spectra of C1s and O1s of pure BC. (**C**,**D**) TGA and DSC analysis from 3-aminopropyl triethoxysilane treated BC membrane (BC-APS), vinyl-triethoxy silane treated BC membrane (BC-VS), acrylated BC membrane (BC-AA), acetylated BC membrane (BC-AC). Inset (**C**,**D**): initial degradation step. Reproduced with permission from [41] (Copyright © 2022, Elsevier), [37] (Open Access).

X-ray Photoelectron spectroscopy (XPS) of the BC material gives evidence of the chemical state of the material. Two sharp peaks are observed in the region of 288 and 534 eV which belongs to the C1s and O1s spectra in the XPS spectra of pure BC [41]. By deconvoluting the peaks, we can obtain information about the carbon and oxygen bonding. The C1s (Figure 3A) contains 4 peaks, first one belonging to non-functionalized carbon –C–H, C–C, the second peak can be designated to C–O, third can be designated to two non-carbonyl oxygen atoms O–C–O and carbon atom attached to single carbonyl oxygen C=O, fourth is the carbon attached to a carbonyl and non-carbonyl oxygen O–C=O. However, the O1s peak (Figure 3B) is deconvoluted into three peaks assigned to the O–H, C–O–C and O–C–O group and the third peak can be assigned to –CONH_2_. Energy dispersive X-ray spectroscopy (EDS) is another way of analyzing the distribution of elements in the matrix of BC.

Thermogravimetric analysis (TGA) helps in the evaluation of the stability of synthesized BC which and gives a good understanding of further modification of the bio-polymer [42]. It has been reported that synthesized BC has a better stability then the commercial ones [37]. The TGA graph (Figure 3C) shows three distinct wight loss region. The first was contributed by free and bound water molecules within 150 °C with a total 5% weight loss, The major weight loss peak was observed in the differential scanning calorimetry (DSC) graph, (Figure 3D), which is the first derivative of the weight loss curve, between 200–400 °C and can be attributed to the depolymerization, dehydration and decomposition processes of the framework with a 75% weight loss. The peak due to pyrolysis of cellulose is observed as a shoulder to the right (Figure 3D). The final residue was 15% of the original weight and was charred BC. The features of the glass transition temperature (T_g_) using pure, and surface treated BC, were similar. As we take a closer look in the region of 50–200 °C we observe a change in the onset thermal degradation temperature (T_o_) due to surface modification. In addition, the surface modifications can lead to increase in the maximum degradation temperature (T_d_). However, the surface modified by silane showed the maximum improvement to 10 °C and gave the highest residue. In between 80–140 °C the transformation is reported to be the melting of the crystalline phase, after which the BC has stability till it starts decomposing at 350 °C where the maximum transformation is observed [43].

Mechanical response of BC using an in-situ nano-indenter with BC under aqueous state has been studied in details [21,22]. It was deduced from the *P-h* curve obtained that the mechanism of inelastic/pseudoplastic deformation of BC was independent of the loading rate as well as the peak indentation and shows an absence of residual stress even with higher degree of penetration depth by the indenter. Loading–unloading nano-indentation tests were performed with a Berkovich indenter using a multi-module mechanical tester where-in the contact area as a function of indenting depth is calibrated using fused silica and tungsten as reference samples at room temperature in the liquid cell where the samples are submerged. From the load applied and with the displacement in the indenter, a plot of load vs. depth was used to find the hardness and the Young’s modulus of the BC sample. From this setup, the creep deformation profile was also measured from different dwell periods at different peak indentation loads. The modulus and hardness were calculated using the famous Oliver–Pharr method. The Young’s modulus for the synthesized hydrated BC nanocellulose have been reported to be 0.0025 GPa inside the samples up to 0.04 GPa at the surface, the dwell period obtained at 20 s, and tensile strength of 0.05–1.0 MPa, respectively [22]. The exceptionally low value of Young’s modulus was due to its high porosity of BC hydrogel with high viscoelastic behavior. By a simple manipulation of the surface the elasticity of the BC samples could be made comparable to some well-known composite for in vivo application. BC is both flexible and amorphous with effectively lower tensile mechanical properties similar to mechanical features of a hemicellulose structure. Studies have reported BC tensile strength on an average of 241.42 ± 21.86 MPa, an elongation value around 8.21 ± 3.01%, and Young’s modulus of 6.86 ± 0.32 GPa [44].

The wettability of the BC is also an important feature of the scaffold used for adhesion of cells by measuring static water contact angles and the water drop profiles, which has proven to be hydrophobic and is imperative for the anti-bacterial and anti-fungal properties [20,21,45]. The dynamics involving the adhesion of the beneficial cell while eliminating the bacterial or fungal growth on the surface of BC is vital for the use of such material in living body. Scanning electron microscopy (SEM) (Figure 1B), atomic force microscopy (AFM), transmission electron microcopy (TEM) and confocal microscopy (CM) are types of microscopies which has been widely used to study the morphological changes and the topography of BC material. Using these microscopies, we obtain information on the surface characteristics and how the surface affects cell growth and adhesion. BC has a broad spectrum of properties which make them a promising biomaterial for various biomedical applications [18,20,21,45].

## 3. Functionalization of BC Hydrogels

**Figure 4:**
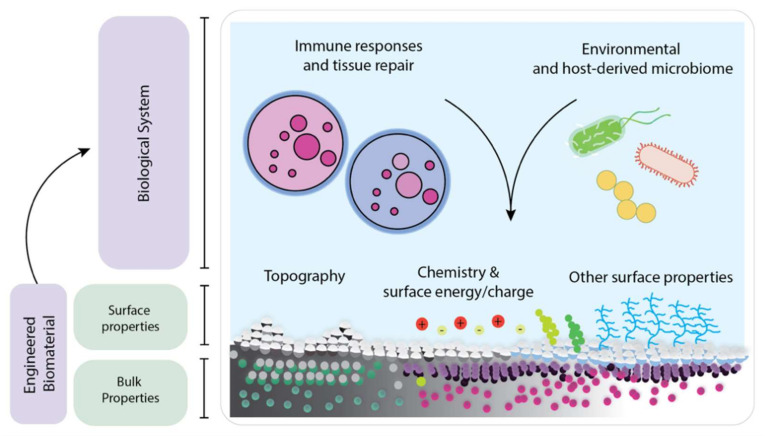
Schematic showing the ideal biomaterial as a combination of engineered bulk and surface properties that trigger adequate immune responses while minimizing the risk of infection, commonly referred as “the race for the surface”.

When a biomaterial is placed in the body, its surface is the first to contact various substances and agents, including growth factors, proteins, immune cells, as well as foreign and host-derived microbiome. Water or other liquid retaining capacities of the polymer hydrogel provides the vessel for loading liquid drugs and bioactive compounds into the vicinity of the dressing material. Thus, the surface characteristics play a crucial role in determining the performance of a biomaterial [46]. Surface properties such as topography, wettability, surface charge, and chemistry can directly influence the cell-surface interactions through the transduction of biomechanical stimuli into chemical stimuli, resulting in the activation of different signaling pathways which guide different cellular responses such as the integration of the biomaterial with the host tissue, appropriate immune responses, and infection prevention [47,48,49,50,51,52,53,54]. Better surface characteristics gives the possibility of improved interaction with living cells which would then support migration of epithelial cells and fibroblasts to help in accelerated replacement of the lost or damaged tissues resulting in protection of the wound from infection, reducing pain, and lowering health care expenses. An ideal biomaterial should possess the right bulk and surface properties to trigger a desired mechanical performance while inducing an adequate immune response (Figure 4). As mentioned previously, BC has been used as biomaterial in many ways. This is due to its unique bulk characteristics. Nonetheless, surface modification processes that retain the bulk properties are desired because they extend the BC’s capabilities [55]. There are different approaches to modify the BC surface properties. A standard method for surface modification is performed by chemically treating the BC to incorporate functional groups, nanoparticles, and synthetic polymers. On the other hand, plasma and irradiation-based methods can modify the BC’s chemistry and physical properties in a single step while retaining its native bulk properties. We will describe each of those methods in greater detail in this section. It is important to note that plasma and irradiation-based techniques are inherently surface limited processes. Their penetration ability is determined by the penetration of energetic particles, i.e., ions, electrons and radicals, into a solid material. For particles in the 10^2^ to 10^3^ eV energy range, the particle penetration is typically in the 10^0^ to 10^1^ nanometers. Similarly, light irradiation processes can be limited from microns to a few millimeters in depth. Their surface limited characteristic is directly determined by the wavelength (energy) of the incident radiation [56].

### 3.1. Chemical Methods

The chemical-based treatment takes advantage of the active sites found on the BC polymeric chains, such as hydroxyl groups. Typically, these chemical methods are performed in an aqueous environment, where the functional groups are activated to then interact with other reactants in the solution. This loads the BC surface with chemically grafted or physically decorated moieties with new functional groups. Chemical modification methods can improve the intrinsic properties of BC membranes such as antibacterial features which proves beneficial for chronic wounds, diabetic ulcers and burns [30]. The different approach to achieve modification of BC can involve (1) substitution in the OH group of BC with other functional groups (2) crosslinking with other polymer materials (3) composite with metal/metal chalcogenide nanomaterials (4) carbon based nanocomposites [57].

**Figure 5:**
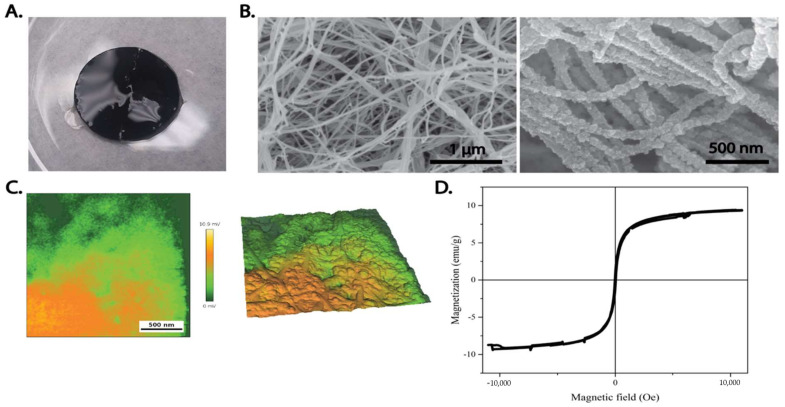
Magnetic BC. (**A**) Macroscopic appearance of a magnetic BC hydrogel loaded with ferromagnetic nanoparticles. SEM images of (**B**) Pristine (left) and (**C**) magnetite-functionalized BC (right). (**D**) Magnetization saturation curve for 100 mM MBC shows that composite is superparamagnetic and has a maximum magnetic saturation of 10 emu/g. Reproduced with permission from [18] (Copyright © 2022, Journal of Visualized Experiments), [21] (Copyright © 2022, Elsevier).

(1) Substitution of BC at the -OH group is a common method to obtain oxidized, amidoximated, acetylated, acrylated and phosphprylated BC derivatives. Specific surface properties can be imparted in the modified BC by carefully choosing the reactants to be chemically grafted. For instance, functional groups with well-known biocidal properties, i.e., ammonium [58] and aminoalkyl groups [59], have been used to enhance the non-existent antibacterial capabilities of BC. Again, the surface acetylation of BC, in freeze drying processes, yields membranes with higher drug retention and improved drug release rates compared to pristine ones. This is achieved by a combination of swelling of the BC induced by the freeze drying, which enhances the uptake capabilities of the membranes, and improved hydrophilicity due to the acetylation. The combined effects leads to drug retention deeper into the membranes, which derives its higher retention [60]. These derivatives can be further used for attracting nanoparticle composite of BC.

(2) Polymer both natural and synthetic with complimentary structures, can be crosslinked by covalent or non-covalent linking or physically attached composites with BC have been established with enhanced characteristics [61,62,63]. Some natural polymers such as chitosan, gelatin, collagen, sodium alginate, hyaluronan, carrageenan have been commonly used for such purpose [27,64,65].

(3) Metal or metal chalcogenides are fabricated to yield nanoparticle decorated BC nanocomposites [66,67,68]. The advantage of nanocomposite materials is that they combine the properties of two or more different materials that can excel the mechanical properties and functionalities of the components alone [66,69,70,71]. Nanocomposites formed by the integration of NPs into BC are of great interest because they offer a wide range of applications (Figure 4) [72]. Due to their characteristics and preparation process, light-sensitive NPs can be synthesized for sensing, imaging and radiotherapy [73,74,75]. The nature of the NPs can also make them an attractive solution for drug delivery purposes, owing to the presence of functional groups and encapsulation capabilities [76]. Functionalization of BC with magnetic nanoparticles have been proposed for accelerating the healing of damaged blood vessels in conditions such as brain aneurysms, which still face high fatality rates Figure 5.

(4) Carbon based materials like carbon nanotube, graphene, etc., have been frequently utilized to make composites with BC. These composites possess good conductivity and mechanical strength and is frequently used in electronics and biosensors [77,78,79,80,81,82].

Typically, chemical modification of the BC pellicles is performed to further improve the nanocomposite integration by chemically grafting functional groups on the hydroxyl ends of the BC chains before the nanocomposite synthesis, which improves the adhesion of the different phases comprising the material [83,84]. Preparation methods may include sterilization, dehydration, and pH adjustment of the solution where the synthesis occurs [85,86,87].

### 3.2. Plasma Irradiation and Other Irradiation Methods

A different approach to produce surface-modified BC relies on treating the pellicles with photons or energetic particles. These irradiation-based processes are attractive because they eliminate the need for additional reactants in the treatment. Ion irradiation processes offer a unique approach to the surface functionalization of BC. This approach modifies both the inherent chemical and physical properties of the BC surface with high fidelity. In particular, directed irradiation synthesis (DIS) or directed plasma nanosynthesis (DPNS), where energetic ions are extracted from low-temperature plasma and combined in unique ways, can induce significant functional changes to BC materials such as physical nanopatterning or chemical bond transformation without inducing damage. In addition to this, ion irradiation also triggers the synthesis of new agents following simple chemical pathways. A remarkable fact about the ion irradiation process is its ability to simultaneously drive the surface modification and synthesis of the functionalizing agents without additional chemical processes. Ion irradiation is a technique that can produce advanced BC-based biomaterials in a single step, green chemistry, sustainable manufacturing approach.

Laser irradiation is a simple technique that allows producing patterns on the BC’s surface while preserving its chemical and crystalline properties. For example, laser irradiation has been used to introduce micro scale pores on BC pellicles, thus improving cellular ingrowth for tissue engineering scaffolds [88]. In the domain of energetic particles, electron beam irradiation has also been used to fabricate stimuli-responsive hydrogels based on BC. Under irradiation with electrons, hydroxyl radicals generated by the radiolysis of water and the breakage of polymeric chains lead to the formation of active sites in the BC that subsequently recombine with other macroradicals to form highly cross-linked structures. These cross-linking reactions play a key role in grafting synthetic polymers like acrylic acid to BC without the use of cross-linking agents, which depend also on the electron dose. Electron beam-induced crosslinking is accompanied by morphological changes in the BC pellicles at the microscale. The most notable effect is the change in the BC’s pore size, and thus, changes in its crystallographic properties [89,90]. However, highly cross-linked porous BC membranes exhibit increased swelling capabilities and improved biodegradability when compared to their non-irradiated counterparts [91]. These features make them great candidate materials for drug delivery applications [92]. The main drawback of electron beam treatment is that the reported improvement of the biological properties of BC is attained with irradiation with high energy electrons (in the order of MeV) and high doses (in the order of tens to hundreds of kGy). Irradiations of these characteristics require large-scale facilities, i.e., particle accelerators, which can incur an increased cost of potential product commercialization.

Compared to high-energy high-dose electron beam treatment, plasma treatments require significantly simpler setups, including a vacuum vessel, a plasma source to generate the discharge, gas sources to feed the plasma, and the vacuum generation equipment, i.e., vacuum pumps [41]. Plasma treatment can be performed using noble or reactive gases. Regardless of the species used, BC membranes undergoing plasma treatment exhibit significant changes in surface morphology. For example, plasma treatment induces the disruption of the BC’s fibrous matrix, typical of pristine pellicles. This transformation takes place due to fiber breakage and aggregation. Plasma treatment also augments the roughness of BC with increasing plasma treatment times. The physical transformation of plasma-treated BC is also accompanied by chemical changes, which are characterized by alterations in the atomic composition, chemical states, or density of the functional groups on the surface [93]. For example, by carefully selecting the plasma species and treatment conditions, the functional group density at the BC’s surface can be controlled, leading to the production of either hydrophobic or hydrophilic membranes [41,93,94,95].

**Figure 6:**
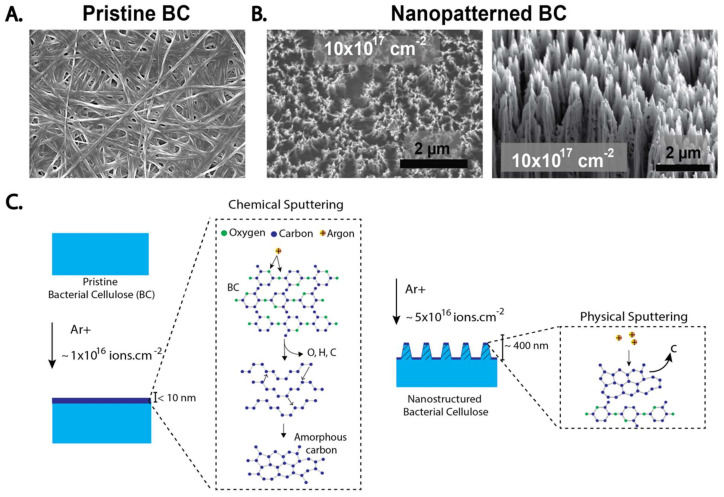
SEM images of (**A**) pristine BC (**B**) irradiated BC. (**C**) Chemical and physical sputtering of BC surface. Reproduced with permission from [19] (Copyright © 2022, American Chemical Society).

An example of ion irradiation-based surface modification technique uses ion bombardment to drive the surface away from equilibrium. This approach is advantageous because it uses exclusively energetic ions, allowing for the synergistic physical and chemical modification of a material with high fidelity and controllability [96]. For example, BC subjected to ion-irradiation leads to the formation of self-organized nanostructures by reorganization of the fibrous surface structure (Figure 6). In addition to this morphological transformation, ion irradiation of BC also induces chemical changes, including alteration of the atomic composition and crystallinity [19]. The properties of these nanopatterned surfaces make ion-irradiated BC a great candidate as a biocidal surface [20,97]. In most cases, the nature of the irradiation treatments allows to preserve the nature of the bulk BC chemistry. This is because the interaction of polymers with energetic particles, neutrals or photons drives specific responses on the surface of the treated material. These include surface activation, surface etching, chain scission and cross linking. Nonetheless, the surface activation can be used as a platform for grafting or film deposition on irradiated BC. This makes irradiation-based processes an attractive alternative to chemical treatments [98,99].

**Figure 7:**
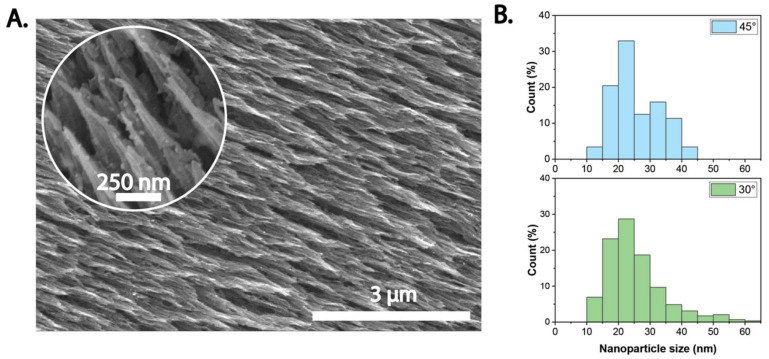
Silver-loaded nanopatterned BC fabricated via ion beam irradiation. (**A**) SEM images, and (**B**) nanoparticle size distribution at two different irradiation angles.

The ion irradiation approach has also exhibited the capability to produce surface modified and functionalized BC. By adding a precursor, silver nanoparticles-decorated nanopatterned BC has been fabricated (Figure 7). This has been achieved by a single step process, in which the energetic ions drive the surface morphology modification and the synthesis of silver nanoparticles, simultaneously. These unique functionalized surfaces combine the bactericidal capabilities of nanopatterned surfaces [20,100], with the well-known antibacterial properties of silver nanoparticles. Although, control of the ion irradiation over the characteristics of the nanopatterned surfaces is better understood, the tunability of the silver nanoparticles size and properties remains to be further explored. Moreover, the use of other precursors to fabricate different metallic nanoparticles is promising.

## 4. Biomedical Applications of Functionalized BC Hydrogels

Since these products of BC contain low levels of endotoxins (<20 EU per device), they are FDA approved for their usage as tissue replacement products [27]. BC composites have a varied number of application, not only in biomedical, but also in cosmetic, food and additive manufacturing. Below are examples of some of the popular use of BC primarily for biomedical application as provided in Table 1 [61,62,77,101,102,103].

### 4.1. Vascular and Neural Applications

Degradation of cellulose occurs by hydrolysis, but the compact structure of BC is highly resistant to degradation. Due to lack of the enzyme cellulase in the human body, an enzyme that hydrolyzes the β-1,4 D-glucose linkages of BC, making it a well-suited biomaterial for vascular, conduit and neural implant [21]. This tunable biodegradability of BC is suitable for using it as a material for implants. One of the critical concerns of vascular implants is thrombosis and occlusion which has led to the search of non-thrombogenic material with comparable mechanical property especially anisotropy for native blood vessels. The moldability of BC was advantageously used for making of vascular tubes and was patented under the name “BASYC” [104]. The molds could be stored for 6 weeks at 4 °C without any degradation. Investigating BASYC with controlled blood vessels of rat proved that the inner surface was comparable ranging from 7–14 nm, tensile testing provided load capacities of mean value 800 nm, the tubes sustained a blood pressure of 0.02 MPa, and had a 100% patency rate with no rejection signs after weeks. The tubes were well traversed by connective tissue and small vessels, and was layered with endogenous cells [105]. In another study, a typical artery vessel which consists of three concentric layers of intima, media, and adventitia was successfully mimicked [106]. BC laden with three layered cells like a simulated blood vessel showed a steady proliferation of the cells with the development of filopodia and lamellipodia with high level of cell viability. Longer period of cell culture produced Collagen I which held the BC fibers and cells together leading to high cell compatibility. The technique used to monitor in vivo performance of BC is Doppler ultrasound imaging. From in vivo studies neither thrombus nor immune response around the BC was detected.

The bending stiffness of central nervous system and peripheral nervous system should be near the Young’s modulus of the native tissues of brain and peripheral nerve which is 2.7–3.1, and 580–840 kPa, respectively, which is in compliance with the Youngs’s modulus of BC around 80–120 kPa and is less rigid to other conventional polymers. Neural oriented research establishes the usability of BC to enhance nerve cells [17,22]. BC has a porous structure which in the hydrated form contains about 99% percentage of water and this features the extracellular matrix of tissues. There are numerous reports of cellulose-based electrodes and conductive material. The bending stiffness of the electrodes depends primarily on the width of electrodes and that of a single channel, which can be toned down to as low as 5 μm, retaining good conductivity and surface roughness for neural implants and hence more at par with the brain and nerve tissues [42]. This is compatible with single mammalian cell, which is between 10–100 μm. Their thin structure also makes it foldable and easy to bend and attach to surfaces. The well transparent hydrated BC containing electrodes is required for transmittance analysis for locating the interface sites in vivo and study the morphological changes occurring in the tissues beneath the electrode. The BC electrodes show commendable durability and reliability when subjected to fatigue test showing very low increase in resistivity despite considerable cycles.

Neurodegenerative impairment due to cervical spinal cord injury, Parkinson’s disease and Alzheimer’s disease cause irreversible damages to neurons. There are challenges involving application to human body such as functional engraftment and uncontrolled differentiation of implant. The use of BC scaffolds in neural stem cell-based therapy (NSC) for neuron restoration and repair of damaged cells may be achieved by designing appropriate spatial and temporal NSC microenvironment with scaffolds which effects the physiological stimuli around the NSCs such as culture media, surrounding cell co-culture, physicochemical parameters, surface chemistry, topography, and mechanical signals [53]. This type of 3D porous conductive scaffold has given a new dimension to BC-based material design where such porous microenvironment can nurture neurogenesis and control cell behavior with high-fidelity [17,22]. Electrical functionalization of BC can be favorable for a wide range of application including, electrically induced drug delivery, biosensors, vascular repair and bioelectronics [107].

### 4.2. Wound Healing

There is another biomedical area where BC has made an impact and that is wound healing of organs in the human body. In USA, approximately 2% of the population could be affected by chronic wounds. Medicare in 2018 reported cost estimates for acute and chronic wound treatments of about USD 28.1 billion to USD 96.8 billion. The investment in the wound care market is expected to exceed USD 22 billion by 2024 driven by the development of new wound care products, increased incidence of chronic wounds and government support [108]. The skin is the largest organ in the human body. Its three-layered structure maintains the internal homeostasis and protects the body from the external environment and possible pathogens. Trauma, systemic diseases, and changes in nutrition can alter the integrity of the skin, which can be restored via the wound healing process. However, some conditions like burns or extensive injuries can slow down skin regeneration and cause chronic inflammation [26]. Dressings in the form of films, foams and hydrogels from different materials are commonly employed as wound coverages for these cases, yet they are susceptible to bacterial infections [109].

Skin cancer and serious burns are also treated using dressings [110]. In the United States non-melanoma skin cancers (5 million people per year), melanoma skin cancers (280,000 people per year) and serious burns (486,000 people per year) require dressings that shorten the wound healing process to prevent inflammation and microbial infection, and in the case of cancer it needs to prevent tumor recurrence as well [26,111,112,113]. The ideal wound dressing, thus; must accelerate reepithelization, maintain wound moisture, oxygen permeability, reduce pain, prevent infection and be customizable [26]. BC microfibrillar structure serves as a 3D flexible scaffold, which can act as a physical barrier against pathogens, while promoting cell attachment and tissue granulation. Its structure allows it to retain large amount of water, which keeps the wound moist, promotes gas exchange and absorbs exudates from the injured tissue. In addition, its high levels of innate purity makes it biocompatible, non-toxic and non-allergenic [26,27,114].

BC is FDA approved for wound dressing and has been widely commercialized. Biofill^®^ was the first BC film used as a skin substitute on the market. It has been used to treat skin carcinomas, severe burns, dermabrasions, and chronic ulcers. These films adhere closely to the wound bed but detach after reepithelization, which result in immediate pain relief. Although these films reduce treatment times and are inexpensive, their low elasticity prevents mobility in some areas. BC has also been used as dry membranes to treat lower limbs chronic varicose ulcers, resulting in superficial wounds in 80% of the patients after 120 days. These membranes induced tissue remodeling and granulation from the central area and margins of the wound. Commercially, XCell^®^ has also been used for this application. It is a never-dried BC wound dressing that maintains wound moisture, promotes autolytic debridement, and accelerates tissue granulation. Bionext^®^ and Bioprocess^®^ are commercial BC products used to treat ulcers and burns, minimizing pain and infection rate. These dressings along with Membracel^®^ and Dermafill^®^ BC membranes have been shown to accelerate wound healing [112,115,116]. The BC functionalization can be enhanced with active ingredients or via physical and chemical methods [112]. For example, using PDMS soft templates microgrooves (≤10 µm) on BC membranes can modulate keratinocytes and fibroblasts attachment, alignment, migration, and proliferation. In addition, it accelerated the wound healing process in vivo after 21 days by limiting the immune response and promoting tissue repair [48,117]. Low-energy carbon dioxide laser lithography has also been used to micropattern the surface of BC with grooves and columns for wound healing applications, modulating cell attachment and migration, and collagen production in vitro which causes low scarring in an in vivo wound healing model [118]. Chemical modifications include creating composites with other polymers like silk-sericin, chitosan, dextran and collagen. These reinforces BC structure, improving its mechanical properties and accelerating the wound healing process [57,112,119,120,121].

BC can be used as a drug delivery system to manage diseases. For example, it has been used to control the delivery of curcumin to improve tissue granulation, in addition to its antifungal, anticancer, antibacterial and antioxidant properties [77,122]. It has been used to deliver lidocaine to promote tissue repair in third degree burns in rats [123]. As well as a vehicle to deliver antibacterial and antiseptic agents. These antibacterial agents include the antibiotic fusidic acid [124], short-acting drugs like tetracycline to prevent bacterial growth, octenidine, povidone-iodine and quaternary ammonium compounds, which are thought to damage bacteria’s membrane among others [26,58,125,126,127,128].

There are myriads of membranes and barriers that delimitate external surfaces and internal cavities, forming the epithelial tissue. Some of these are the eardrums, cornea, mouth and urogenital conducts, among others. The loss of these tissues could be treated using BC derived materials. For example, for corneal disease that affects approximately 10 million people worldwide, BC is easy to manipulate to create complex structures and promote the growth of human corneal stromal cells in rabbits, making an ideal candidate for this application. However, research in this topic is still in its infancy, requiring more investigation in how to improve BC transparency and elasticity for this application [112,122,129]. It also provides an innovative, effective, safe, minimally invasive and low-cost alternative to close tympanic membrane perforations, commercially BC Gelfoam™ has been used for this application [130]. For urethra tissue engineering, BC scaffolds mimic the mechanical properties of the urethra’s extracellular membrane. Although these membranes have been successfully implanted and integrated in the urethra wall of Wistar rats, remodeling and strengthening it, the compact structure of BC might limit cell migration and ingrowth, requiring alternative strategies [129].

### 4.3. Bactericidal Nanostructures

In vulnerable populations such as elderly and immunocompromised patients, biomaterials and biomedical devices directly contacting tissues and organs increase the risk of nosocomial infection development. These infections lead to the failure of implanted devices and contribute to life-threatening conditions such as bacteremia, which are difficult to treat by conventional systemic antibiotic therapy [131]. Critical to the onset of device-associated infections is the formation of biofilms at the biomaterial interface. Biofilms are multicellular aggregates of bacteria, encased by both host-derived macromolecules and extracellular polymeric substances (EPS) of microbial origin [132]. Bacteria in biofilms are better equipped to cope with external conditions and usually remain refractory to the treatment with antimicrobial compounds and drugs. Indeed, the most effective way hitherto to treat biofilms is to remove the biomaterial, which is particularly traumatic for permanent internal prostheses and other biomedical devices [133].

**Figure 8:**
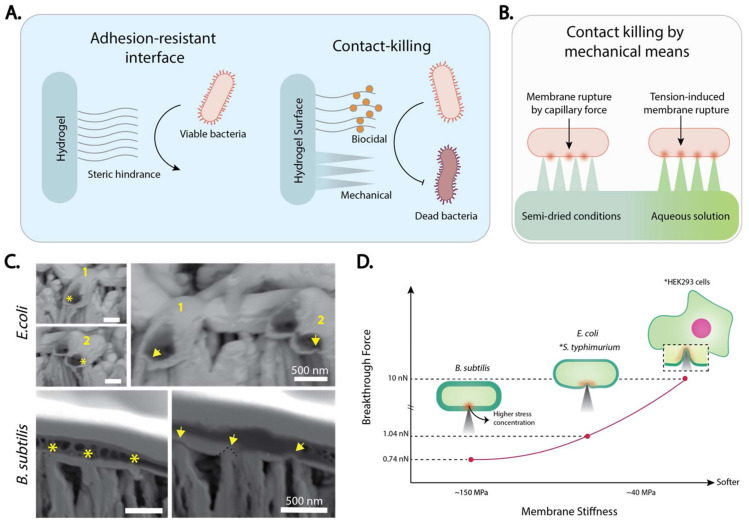
Bactericidal nanostructures fabricated in BC. (**A**) Typical antibiofouling and antimicrobial strategies implemented on hydrogels and very compliant materials. (**B**) Contact-killing via mechanical means can be the result of capillary forces in the air-liquid interface as well as tension-induced mechanical rupture. (**C**) Indentation of the bacterial envelope in Escherichia coli and Bacillus subtilis in contact with nanostructured BC. The asterisks and arrows indicate the indentation left on the bacterial envelope by the BC’s nanostructures before and after the cross-sectional cut, respectively; (**D**) average force values necessary to penetrate *B. subtilis, E. coli, S. typhimurium*, and HEK93 cells as a function of the membrane stiffness. Reproduced with permission from [20]. (Copyright © 2022, American Chemical Society).

Several strategies have been implemented to prevent the undesirable presence of biofilms in hydrogels and very compliant materials. These include adhesion-resistant hydrogels and contact killing strategies that rely on the release of biocidal compounds and biophysical mechanisms (Figure 8A). Adhesion-resistant hydrogels include natural and synthetic polymers (e.g., polymeric brushes based on poly ethylene glycol, that strongly interact with water molecules, preventing protein and bacterial adhesion by steric hindrance). However, those materials are eventually colonized over time, given the extensive repertory of strategies used by bacteria to stick to surfaces, including the secretion of EPS and the use of cellular appendages like flagella and pili for adherence [134,135]. Conversely, contact-killing interfaces aim to prevent biofilms formation by killing bacteria that encounter the material by either releasing biocidal compounds or mechanically disrupting the bacterial envelope through topographical cues. Although biocidal compounds such as nanoparticles and antimicrobial drugs can effectively prevent biofilm formation, they can also be detrimental to the surrounding tissue, they might accumulate in organs like the liver and spleen, and contribute to bacterial resistance by releasing suboptimal antimicrobial doses over time [136,137].

Contact-killing via mechanical pathways takes advantage of the forces exerted by high aspect ratio nanostructures such as nanopillars and nanospikes on biological membranes, which cause their stretching and eventual failure. This bactericidal mechanism was initially observed on the wings of insects like cicadas and dragonflies, as well as on the gecko skin. Compared to biocidal compounds, bactericidal topographies do not contribute to bacterial resistance, nor consumed metabolically, and are not harmful to the surrounding tissue [138]. Current evidence indicates that at least two different forces contribute to the observed bactericidal mechanism: one driven by capillary forces that appear under semi-dried conditions like those induced by hydrophobic interfaces [139] and a tension-induced membrane rupture within the activation energy theory that occurs primarily on pristine hydrophilic materials [20] (Figure 8B). Very recently Allain etl.al fabricated bactericidal nanostructures in BC using low-energy ion beam irradiation with argon ions and demonstrated that thicker membranes, like those in Gram-positive bacteria, are more susceptible to membrane rupture than those in Gram-negative bacteria, which possesses a thinner membrane (Figure 8C,D) [20]. These observations are consistent with a large body of evidence showing higher susceptibility of thicker membranes to mechanical disruption upon contact with single-walled nanotubes and graphene nanosheets, breaching the gap between the bactericidal mechanism of low dimensional materials and bactericidal topographies [20].

### 4.4. Other Biomedical Applications

BC has also been used as biosensors, cosmetics and in bone tissue engineering applications. BC is non-toxic, has good mechanical properties and has an interconnected porous structure, which makes it ideal for bone tissue engineering applications. Composites with other materials like multiwalled carbon nanotubes, collagen, hydroxyapatite, gelatin, silk fibroin, paraffin wax and graphene oxide has been designed to modulate the scaffold porosity and its biological and mechanical properties [140,141,142,143,144,145,146,147,148].

Since BC can be easily modified and functionalized with nanoparticles, carbon nanotubes, metal oxides, conductive materials, and biomolecules, it makes BC an interesting material for biosensor applications. These biosensors can transduce electrochemical signals, optical signals and mechanical signals [63,149,150]. In the biomedical field, BC has been used to detect lactate via a lactate oxidase immobilized BC-Prussian blue nanocubes [151], glucose using a glucose oxidase immobilized BC-gold composite and a BC-cadmium telluride quantum dot composite, dopamine using BC-palladium nanoparticles, Nitric oxide and humidity using piezoelectric BC-Quartz crystal microbalance sensors [149], bacterial attachment using a BC-polypyrrole-TiO_2_-Ag nanocomposite [152], BC/polyaniline/single-walled carbon nanotube composites [150], and bacteriophage immobilized on a BC- carboxylated multiwalled carbon nanotubes as polyethyleneimine sensor [50,153].

BC products could also be used in the cosmetic industry as skin drug delivery products. BC’s porous nanostructure, membrane-like shape, mechanical properties and elasticity makes it an ideal candidate to fabricate easy-to-handle, adhesive facemask which can encapsulate antioxidant, anti-inflammatory, antiallergic, anticellulite, antiaging, skin-whitening active ingredients, such as bamboo extract, retinoid, tea polyphenols, ascorbic acid, propolis, caffein, bee venom, hyaluronic acid, among others. This biopolymer in conjunction with other polymers like poly (vinyl alcohol), poly (ethylene glycol diacrylate) and poly (ethylene oxide)-b-poly(caprolactone) nanoparticles can be used to control the release of these substances, improving the mechanical properties and flexibility of the membranes. BC can also be used as a green alternative to microplastics in exfoliating solutions or as surfactants in emulsion stabilizations due to its intrinsic rheological and structural properties [101,154,155].

In the field of additive manufacturing, bacterial cellulose with poor solubility due to its hydrogen bonding limits has application as a bio ink. However, a BC/alginate composites can be extruded or printed (stereolithography) and crosslinked to successfully generate cartilage tissue engineering applications [156,157]. This field is being explored extensively.

## 5. Cost Analysis of BC Production and Commercialization

BC is a material which requires an extensive method of syntheses. This includes expensive equipment and prolonged standing time for its syntheses. Some of the main limitations in using bacterial cellulose in biomedical applications are the high cost of bacterial cell culture media and the low productivity of some bacterial strains. In total, 30% of the cost is associated with the media used for fermentation. Bacteria uses carbon derived from glucose, sucrose, fructose, glycerol, mannitol, and arabitol to synthetize cellulose as well as complex nitrogen and vitamins [36,158,159]. However, carbon sources could be obtained from other resources such as agricultural and industrial waste products, sugarcane molasses, fruit juice and brewery wastes. Some of these sources include wheat straw, spruce hydrolysate, corn stalk hydrolysate, rice bark hydrolysate, wood hot water, wastewater of candied jujube-processing industry, biodiesel and confectionery industry, acetone-butanol-ethanol fermentation, cotton-based textiles, thin stillage, dairy, corn steep liquor, potato peel, banana and orange peel, pineapple, watermelon, orange, muskmelon juice, coconut and Japanese pear juice [158,159,160,161,162]. The addition of certain additives could enhance the yield, such as ethanol, acetan, agar, sodium alginate, xanthan, carboxymethylcellulose, acetic acid, lactate, lignosulfonate, polyacrylamide and yeast extract [114,160]. Despite the high yield with these additives some might affect BC crystallinity and porosity, changing its mechanical properties [114,158,160]. Other alternatives include using cell-free systems via enzyme catalyzed reactions or genetically modifying the strain [36,60,160].

To maximize bacterial cellulose production for industrial applications, it is important to optimize the fermentation process conditions and reactor design, such as temperature, pH, oxygen dissociation and agitation speed, among others. The equipments in BC production include a rotating disk reactor which is half submerged and half exposed to air, a rotary biofilm contactor having a series of circular disks, the usage of spin filters and silicon membranes, among others. Although, static cultures do not affect BC’s degree of polymerization, crystallinity nor its mechanical properties, compared to submerged cultures in agitation which also might induce the growth of cellulose-negative strains [114,160,163]. Despite these strategies, further research is needed to categorially replace the current method of production in favor of more industrially friendly and economically feasible methods.

## 6. Conclusions and Future Outlook

BC production is well aligned with the principles of green chemistry and circular economy. Eco friendly syntheses of BC minimizes waste and reuses material together with creating a high market value specially in the medical field, biotextile for bandaging to advanced skin replacement, arterial stent coating, nerve surgery, dura mater prostheses, cartilage and bone implants, artificial blood vessels, hemostatic and dental membrane.

The next generation of BC bioactive materials will include those with surface modification to open a wide range of biomedical applications. BC surface functionalization has proven to be beneficial for not only vasculature, but also for implant material, wound healing and bio-electronic platforms. Composite hydrogels with nanofabrication techniques are being modified to spatially pattern the microscale environment of the cell-scaffold system. BC offers a reliable matrix in the development of high-technological platforms for diagnosis and treatment of a wide variety of diseases. Some hopeful areas of research would be to ameliorate drug-delivery systems to targeted cells, immobilize enzyme and biomolecules for in vivo study and therapeutics. BC material can also be used in economically feasible portable devices for nano-engineered diagnostic sensors, smart skin graft applications, and wound regeneration therapies. The resemblance of BC with non-osseous living tissues, its biocompatibility, biodegradability and mechanical properties also makes BC an attractive material for future biomedical applications. With increase in the aging population and increased risk factors in major diseases such as cancer, diabetes leading to ulcer complications, neural and vascular degradation, there is an increased demand for natural biocompatible biomaterials for regenerative therapeutics and/or organ replacement. BC with nanopatterned and functionalized surfaces is envisioned as a promising material to meet the demand for bioactivated natural hydrogels. The biodegradability through oxidation treatment and bioresorbablity are aspects of BC which are being widely explored for potential application as heart valve, meniscus, and bone biomaterials. In spite of several decades of research on biomaterial surfaces, more is yet to be discovered from a biomedical point of view. Another popular line of research is the nanocomposite formation with BC which along with integration of nanoparticles presents challenges to assimilate properties of the material for advanced applications. While an active line of research focuses on the combination of metal/metal oxide nanoparticle with polymer materials, cellulose remains an attractive alternate choice for such biocompatible polymers in biomedical applications [164]. The surface modification in particular could open pathways for future biosensors and drug delivery systems. The flexibility and biodegradability of BC conductive surfaces could be an avenue to overcome blood–brain barrier and flexible delivery of drugs. The plastic properties of these BC composites make it an ideal material for attachment with curvilinear tissues. Other avenues for BC on clinical diagnostics activity could be as electromyography and electroencephalography sensors.

Despite the promising attributes of BC as a fourth-generation biomaterial, there remain many challenges. Although BC is FDA approved biomaterial, the more complex bioactive properties such as bactericidal functions and nanopatterned structural properties enhancing cell behavior, can be expensive to establish relevant clinical trials to demonstrate efficacy. Further, regenerative medicine and organ replacement strategies also require extensive regulatory trials that may be difficult to finance unless additional high-risk financing is secured. The applications for BC remain vast and early-state research will continue to provide knowledge of its fundamental properties in both in vitro and in vivo settings. BC may become one of the most versatile biomaterials since the advent of biomaterial like polyether ether ketone given its incredible biocompatible and bioactive properties. Due to their similarities with the extracellular matrix, degradation trend, chemistry, and biological performance without triggering toxicity and immunological responses, naturally occurring polymers, derived from renewable resources and microorganisms, are a potential choice for myriads of biomedical application.

## Figures and Tables

**Table 1:** Some commercial BC composites used.

Name	Use	Clinical Conditions
BASYC^®^	Vessel implant	Coronary artery bypass surgery
Biofill^®^	Wound care	Burns
Bioprocess^®^	Artificial skin	Burns
Bionext^®^	Wound care	
XCell^®^	Wound care	
Cellulon^TM^	Binder	Medication applications including non-woven structures
Cellulon PX microfibrous cellulose	Suspending agent	Suspension of particle encapsulated enzymes
CelMat © MG & CelMat^®^ MG	Protective dressings/jackets	Protection for miners from potential burns
Dermafill^®^	Wound care dressing	Burns
Membracel^®^	Wound care dressing	
Gengiflex	Non-resorbable cellulose	Periodontitis
Gore-Tex^®^	Dental implant	Periodontal tissue improvement
MTA protective tissue	Biocompatible implant	Injury and wound care
Securian	Tissue Reinforcement matrix	Tendon repair
Gelfoam™	Tissue repair	Tympanic membrane perforations
